# Incubation period of wild type of SARS-CoV-2 infections by age, gender, and epidemic periods

**DOI:** 10.3389/fpubh.2022.905020

**Published:** 2022-07-27

**Authors:** Chiara Achangwa, Huikyung Park, Sukhyun Ryu

**Affiliations:** ^1^Department of Preventive Medicine, Konyang University College of Medicine, Daejeon, South Korea; ^2^Myunggok Medical Research Institute, Konyang University College of Medicine, Daejeon, South Korea

**Keywords:** SARS-CoV-2, COVID-19, incubation period, log-normal distribution, quarantine, Korea

## Abstract

**Background:**

The incubation period of the coronavirus disease 2019 (COVID-19) is estimated to vary by demographic factors and the COVID-19 epidemic periods.

**Objective:**

This study examined the incubation period of the wild type of SARS-CoV-2 infections by the different age groups, gender, and epidemic periods in South Korea.

**Methods:**

We collected COVID-19 patient data from the Korean public health authorities and estimated the incubation period by fitting three different distributions, including log-normal, gamma, and Weibull distributions, after stratification by gender and age groups. To identify any temporal impact on the incubation period, we divided the study period into two different epidemic periods (Period-1: 19 January−19 April 2020 and Period-2: 20 April−16 October 2020), and assessed for any differences.

**Results:**

We identified the log-normal as the best-fit model. The estimated median incubation period was 4.6 (95% CI: 3.9–4.9) days, and the 95th percentile was 11.7 (95% CI: 10.2–12.2) days. We found that the incubation period did not differ significantly between males and females (*p* = 0.42), age groups (*p* = 0.60), and the two different epidemic periods (*p* = 0.77).

**Conclusions:**

The incubation period of wild type of SARS-CoV-2 infection during the COVID-19 pandemic 2020, in South Korea, does not likely differ by age group, gender and epidemic period.

## Introduction

The incubation period of an infection is defined as the time interval between exposure to the source of infection and the onset of the first clinical signs and symptoms (1). Identifying the incubation period is crucial to determine the quarantine period for persons who might have been exposed to an infectious agent and to assist in the monitoring, surveillance, control, and modeling of the infectious disease (2, 3). Coronavirus disease 2019 (COVID-19) caused by the severe acute respiratory syndrome coronavirus 2 (SARS-CoV-2) was declared a pandemic by the World Health Organization on 11 March 2020 (4), and as of 4 August 2021, over 200 million cases and 4.2 million deaths from COVID-19 have been reported worldwide (5). In South Korea, since the first COVID-19 case was reported on 19 January 2020 (6), a quarantine of 14 days was instituted for all suspected individuals who were exposed to a laboratory-confirmed COVID-19 case (7).

Previous studies in Korea have estimated the incubation period of COVID-19 based only on COVID-19 cases from Busan city during the early stage of the COVID-19 pandemic (8) and using the viral load data in hospitalized patients (9). However, the incubation period tends to be shorter during the early stage of the COVID-19 pandemic due to biases in case ascertainment (10). Although other more virulent COVID-19 variants such as the Delta variant (B.1.617.2) and Omicron (B.1.1.529) have emerged in South Korea (11, 12), having more information about the wild-type COVID-19 is important for the understanding of the complete epidemiological picture of the early COVID-19 pandemic.

Here, we examined COVID-19 patient data after stratification by age, gender, and epidemic period to identify the effect of these factors on the COVID-19 incubation period in South Korea.

## Methods

We used the COVID-19 case data published by the South Korean public health authorities including local and provincial department of public health. As part of the COVID-19 monitoring and evaluation strategy, departments of public health in South Korea collected and published information of all new cases on their webpages. We collected data pertaining to sociodemographic characteristics, including age, gender, and city of residence, as well as information on the date of onset of symptoms (the date reported by the patient on which the clinical symptoms first appeared), travel history, contact history, date of laboratory confirmation, and exposure dates. Patients were independently screened by the health personnel for the presence or absence of COVID-19-related symptoms. To ensure the reliability of our analysis, we included only laboratory confirmed COVID-19 cases with complete available data on the date of symptom onset and date of possible SARS-CoV-2 exposure. A confirmed COVID-19 case was defined as an individual with a positive real-time reverse transcription-polymerase chain reaction for SARS-CoV-2, consistent with the protocol approved by the Korea Centers for Disease Control Agency (7).

We used three commonly used distributions (log-normal, gamma and Weibull) for the incubation period and selected the best-fit model by comparing the Akaike Information Criterion (AIC) values for the three types of models (13, 14). We used the three common distributions of incubation period through the probability density function.

The probability density function for the log-normal distribution, *f*_*LN*_, is given as:


$$\begin{array}{l} f_{LN}\left(x;\mu ,\sigma \right)=\frac{1}{x\sigma \sqrt{2\pi }}\exp\left(-\frac{1}{2}{\left(\frac{\ln\;x-\mu }{\sigma }\right)}^{2}\right),\;x>0, \end{array}$$

where μ is the location parameter (the mean of the natural logarithm of the distribution), and σ>0 is the scale parameter (the standard deviation of the natural logarithm of the distribution).

The probability density function for the gamma distribution, *f*_*G*_, is expressed as:


$$\begin{array}{l} f_{G}\left(x;\alpha ,\;\beta \right)=\;\frac{\beta^{\alpha }}{\text{Γ}\left(\alpha \right)}x^{\alpha -1}\exp\left(-\beta x\right)\;,\;x>0, \end{array}$$

where *a* > 0, β > 0 and Γ are the shape, rate parameter (which is the reciprocal of the scale parameter), and the gamma function, respectively.

The probability density function of the Weibull distribution, *f*_*W*_, is given as:


$$\begin{array}{l} f_{W}\left(x;\lambda ,k\right)=\frac{k}{\lambda }{\left(\frac{x}{\lambda }\right)}^{k-1}\exp\left(-{\left(\frac{x}{\lambda }\right)}^{k}\right),\;x\geq 0, \end{array}$$

where *k* > 0 and λ > 0 are the shape and scale parameter, respectively.

We selected the model with the smallest AIC value as the best-fit model. Then, we considered two epidemic periods, based on the two epidemic waves of COVID-19 in South Korea (Period-1: 19 January−19 April 2020 and Period-2: 20 April−16 October 2020) (15). Due to the nature of the initial COVID-19 dataset which included cases from 18 years and above, we categorized the data into broadly defined age-groups based on review of literature. These age groups were: young adults (18–35 years), middle-aged adults (36–55 years), and older adults (>55 years) (16).

All our statistical analyses for demographic and epidemiological characteristics of the confirmed cases were stratified by the two epidemic periods. The mean, median, and 95th percentile of the incubation period was estimated. Descriptive data are reported as frequency counts and percentages. The Chi-squared test was used to identify any significant differences in the demographic variables, and *p*-values < 0.05 were considered to indicate statistical significance. All analyses were conducted using R software, version 4.0.1 (17).

## Results

A total of 287 confirmed COVID-19 cases were included in this study, with 48.8% of patients being females and 39.4% patients in the age group 18–35 years. We estimated the overall median incubation period of COVID-19 to be 4.61 (95% CI: 3.92–4.85) days. The log-normal model was the best-fit model with the lowest AIC (1,604.0) compared to the Weibull (1,635.4) and gamma models (1,615.8) (Table 1).

**Table 1:** Estimated incubation period for all confirmed COVID-19 cases.

**Parameter**	**Log-normal model**	**Weibull model**	**Gamma model**
Mean (95% CI)	4.72 (3.96–4.85)	3.51 (3.01–3.82)	4.56 (3.94–4.83)
Median (95% CI)	4.61 (3.09–4.85)	4.01 (2.91–4.48)	3.82 (3.11–4.71)
95th percentile (95% CI)	11.74 (10.18–12.22)	10.83 (10.01–11.57)	8.17 (7.95–8.58)
AIC	1,604.00	1,635.39	1,614.84

*AIC, Akaike Information Criterion; CI, Confidence interval; Unit, days*.

**Figure 1:**
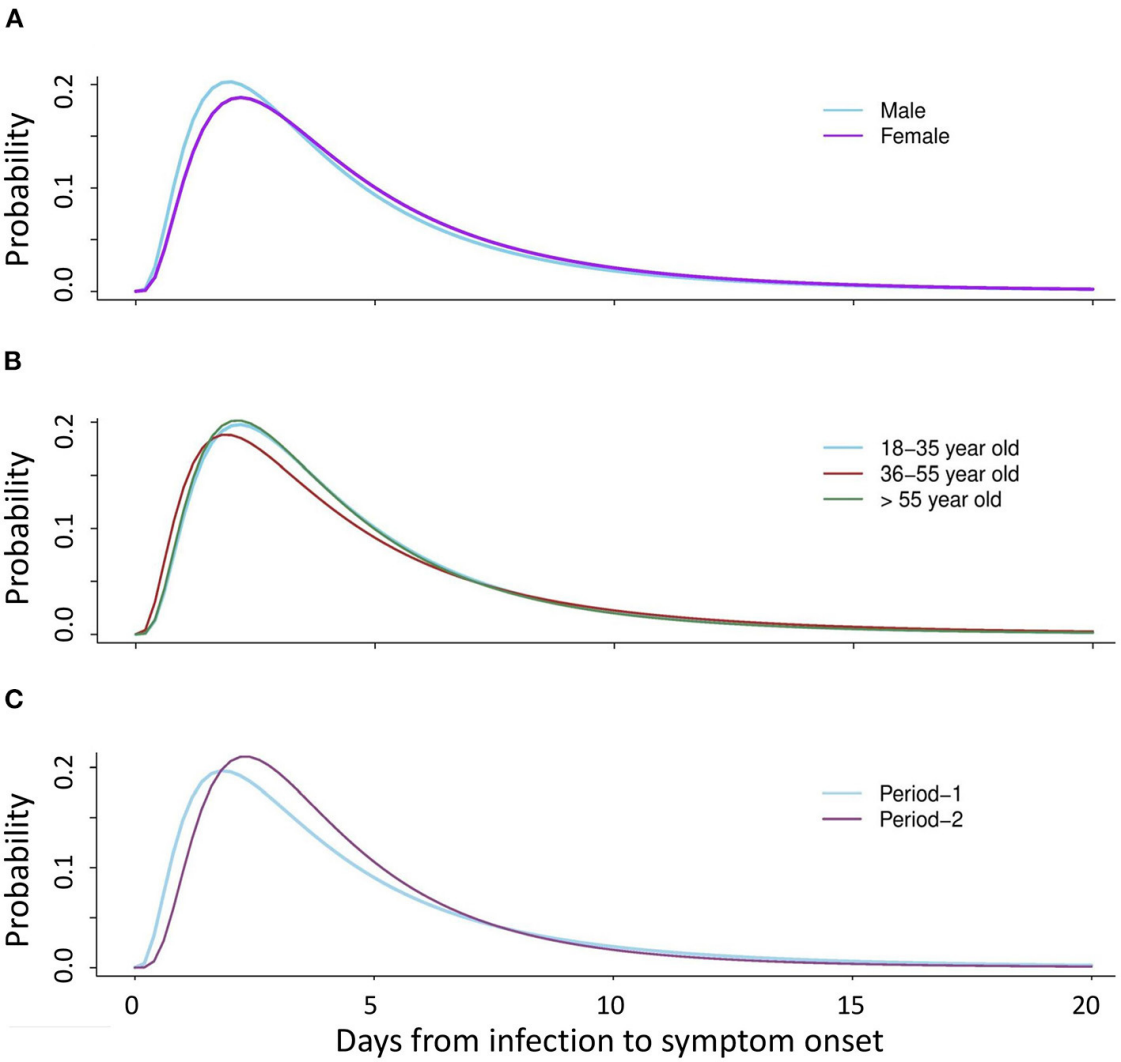
Distribution of the COVID-19 incubation period after stratification by gender, age group, and epidemic period (Period-1: 19 January−19 April 2020 and Period-2: 20 April−16 October 2020) for the confirmed cases using the log-normal distribution model. **(A)** Estimated median incubation period after stratification by gender: male (median: 4.9 days); female (median: 4.6 days). **(B)** Estimated median incubation period after stratification by age: young adults (median: 4.8 days), middle-aged adults (median: 5.0 days), older adults (median: 4.3 days). **(C)** Estimated median incubation period after stratification by epidemic period: Period-1 (median: 4.8 days), and Period-2 (median: 4.4 days).

No significant differences were observed between the subgroups (Table 2). The log-normal model showed the best fit for all subgroups after stratification by gender, epidemic period, and age group (Figure 1). Males had a slightly longer median incubation period of 4.94 (95% CI: 4.41–5.04) days compared to females with 4.63 (95% CI: 4.23–4.79) days, but this difference was not significant (*p* = 0.41). After stratification by epidemic period, the median incubation time for period-1 was slightly longer; [4.81 (95% CI: 4.31–4.99) days] than that for period-2 [4.44 (95% CI: 4.41–4.59) days]. The observed difference, was not significant (*p* = 0.77). Considering age groups, the median incubation period for the middle-aged adults was longer [4.95 (95% CI: 4.39–5.01) days] than that for other age groups [18–35 years: median 4.83 (95% CI: 4.57–4.97) days], 36–55 years: median 4.95 (CI: 4.39–5.01) days, and 55 years and above: median [4.34 (95% CI: 3.96–4.48) days]. However, we found that there was no statistical difference (*p* = 0.60) (Table 3).

**Table 2:** Characteristics of patients with laboratory-confirmed COVID-19 included in this analysis (*N* = 287).

**Variable**	**Total**, ***n*** **(%)**	**Period-1**, ***n*** **(%)**	**Period-2**, ***n*** **(%)**	***p*** **-value**
Age (years)				0.60
18–35	113 (39.4)	64 (40)	49 (38.6)	
36–55	83 (28.9)	49 (30.6)	34 (26.8)	
>55	91 (31.7)	47 (29.4)	44 (34.6)	
Gender^*^				0.97
Female	140 (51.5)	83 (51.9)	57 (50.9)	
Male	132 (48.5)	77 (48.1)	55 (49.1)	

*n (%), number and percentage, p-value: we used the Chi-square test to check for any significant differences, Period-1: 19 January−19 April 2020 and Period-2: 20 April−16 October 2020*.

* *There were 15 entries without a gender allocation*.

**Table 3:** Estimated incubation period for confirmed COVID-19 cases stratified by gender, epidemic period, and age group (years).

**Sub-group**	**Parameter**	**Log-normal model**	**Weibull model**	**Gamma model**
Gender
Male	Mean (95% CI)	4.98 (4.43–5.09)	3.78 (3.33–3.97)	4.59 (4.05–4.71)
	Median (95% CI)	4.94 (4.41–5.04)	4.24 (3.90–4.43)	4.08 (3.90–4.47)
	95th percentile (95% CI)	12.62 (11.69–12.97)	11.11 (10.97–11.49)	11.04 (10.83–11.89)
	AIC	647.50	651.67	681.89
Female	Mean (95% CI)	4.68 (4.25–4.89)	3.55 (3.10–3.91)	4.83 (4.03–4.99)
	Median (95% CI)	4.63 (4.23–4.79)	3.87 (3.11–3.95)	3.81 (3.21–4.0)
	95th percentile (95% CI)	12.14 (10.94–12.87)	11.48 (10.92–11.61)	11.19 (10.86–11.41)
	AIC	673.27	691.57	647.41
Epidemic period
Period-1	Mean (95% CI)	4.89 (4.33–5.03)	3.86 (3.31–3.98)	4.73 (4.22–4.95)
	Median (95% CI)	4.81 (4.31–4.99)	3.96 (3.39–4.01)	3.87 (3.41–4.83)
	95th percentile (95% CI)	13.04 (12.77–13.47)	11.74 (10.45–12.01)	11.63 (9.98–11.99)
	AIC	787.13	800.03	793.97
Period-2	Mean (95% CI)	4.47 (4.45–5.01)	3.82 (3.04–4.01)	4.39 (3.90–4.69)
	Median (95% CI)	4.44 (4.41–4.59)	3.86 (3.33–3.99)	3.87 (3.43–3.98)
	95th percentile (95% CI)	10.57 (10.04–10.97)	10.02 (9.57–10.33)	9.66 (8.64–9.82)
	AIC	814.30	834.48	817.57
Age-group
18–35	Mean (95% CI)	4.88 (4.59–4.99)	4.03 (3.90–4.33)	4.83 (4.10–5.11)
	Median (95% CI)	4.83 (4.57–4.97)	3.95 (3.34–4.11)	4.05 (3.71–4.23)
	95th percentile (95% CI)	12.58 (10.97–12.78)	12.21 (11.97–12.54)	11.66 (10.87–11.81)
	AIC	552.65	571.75	563.20
36–55	Mean (95% CI)	4.98 (4.04–5.19)	3.28 (2.97–3.66)	4.82 (4.01–4.97)
	Median (95% CI)	4.95 (4.39–5.01)	4.32 (3.91–4.57)	4.18 (3.98–4.41)
	95th percentile (95% CI)	12.40 (11.91–12.85)	10.53 (9.79–10.98)	10.85 (10.01–11.20)
	AIC	403.67	404.04	406.17
>55	Mean (95% CI)	4.38 (3.96–4.58)	3.21 (2.99–3.54)	4.28 (3.86–3.76)
	Median (95% CI)	4.34 (3.96–4.48)	3.72 (3.19–4.12)	3.65 (3.01–3.77)
	95th percentile (95% CI)	11.02 (10.8–11.65)	9.97 (9.18–10.07)	9.91 (9.12–10.46)
	AIC	423.25	429.62	425.25

*AIC, Akaike Information Criterion; CI, Confidence interval; Unit: days; Period-1: 19 January−19 April 2020 and Period-2: 20 April−16 October 2020*.

## Discussion

This study was conducted to identify the effect of patient factors such as age and gender and factors related to the time on exposure to SARS-CoV-2 such as epidemic period on the incubation time of COVID-19 in South Korea. It is essential to determine the incubation period of an infection that has become an epidemic and even a pandemic, because it plays a key role to determine the quarantine period for the infectious disease.

Previous studies suggested that shorter incubation periods are more likely to be observed in the growing phase of an epidemic (10). The median incubation period of COVID-19 estimated in this study (median of 4.6 days) was longer than that reported in a previous study (median of 3.0 days) carried out in South Korea during the growing epidemic of COVID-19 (February–March 2020) (8). However, our estimated incubation period was shorter than that reported by other studies conducted in China and Vietnam (5.1 and 6.1 days, respectively) (11, 12). A previous study demonstrated that the incubation period is affected by the level of contact tracing due to missed intermediate exposure events or misperceived exposure times (18). In contrast to China and Vietnam, South Korea has implemented an active strategy to control COVID-19 consistently, involving strict continuous contact tracing and extensive testing to detect COVID-19 cases (15). Therefore, the difference in the control strategy and contact tracing efforts could have led to the difference in the estimated incubation period. Nevertheless, the difference could also be explained by the heterogeneous nature of the case data, biological variations among the different populations, and different variants of SARS-CoV-2 (19).

In our study, the log-normal distribution proved to be the best-fit model for our data because it had the lowest AIC value than the other two models. This was similar to a previous study which found the log-normal model to be the best-fit model (10) and coherent with previous reports that the incubation period of acute respiratory viral infections follows a log-normal distribution (20).

The incubation period did not differ significantly by gender, age group, and epidemic period which is in line with a previous study (8). However, another previous study demonstrated that the mean incubation period for male patients (8.0 days) was longer than that for female patients (4.8 days) (21). Regarding age, another study reported a significantly longer median incubation period for elderly patients over 60 years (7.7 days) than for young patients under 30 years (4.0 days) (22). These findings bring out the possibility that the distribution of the incubation period could be dependent to some extent on gender and age. In addition, the difference in host immunity by age, could result in different incubation periods (22). In addition, we postulate that the elderly could have more recall bias and could potentially ignore early COVID-19-related symptoms and only report later when symptoms become severe. This was similar to reports by a previous study stating that patients with severe COVID-19 disease at onset were older in age than those with non-severe disease (23).

Although the incubation period across different populations for an infectious disease under a given set of circumstances should be similar, we found some differences in the incubation period across the study population. The incubation period is probably affected by factors such as the infectious dose of an infectious agent. However, additional study is needed to demonstrate this.

Although our results provide evidence to support the length of quarantine or active monitoring of exposed persons during the COVID-19 pandemic 2020, longer monitoring periods might be required and justifiable in severe cases (24).

This study is among the first studies on the COVID-19 incubation period stratified by two epidemic periods carried out using pooled data in COVID-19 pandemic 2020, South Korea. Moreover, we used three different models for estimating the incubation period and identified the model that best fitted with our data.

This study has some limitations. First, we conducted a retrospective estimation of the incubation period using already collected data, which could be subject to estimation bias as the COVID-19 epidemic continued growing at the time of writing of this manuscript (10). However, for the latter phase of the epidemic in our study (June–August 2020), the daily number of COVID-19 cases was stable (15). Second, we eliminated some of the extracted data because of incomplete information. Third, using publicly reported data may overrepresent cases with severe signs and symptoms, the incubation period for which may be different from that of mild cases. Fourth, at the time of writing of this manuscript and based on the line list data available to us, complete information about patient exposure and clinical evolution was limited, therefore we could not assess the relationship between exposure and the incubation period.

With the current surge in Omicron cases in Korea, continuous multi-layered interventions including case finding and contact tracing, as well as non-pharmaceutical interventions and booster vaccination will be beneficial for its control (25). We recommend that studies on the COVID-19 incubation period also consider the different COVID-19 variants to understand the transmission dynamics of COVID-19 better.

## Data availability statement

Publicly available datasets were analyzed in this study. This data can be found here: https://github.com/achangwa/south-korea-covid-19-data.git.

## Ethics statement

This study was approved by the Konyang University Institutional Review Board (KYU 2021-08-028). Written informed consent for participation was not required for this study in accordance with the national legislation and the institutional requirements.

## Author contributions

SR designed the study. CA and HP collected the data, conducted analysis, and wrote the original draft of manuscript. CA and SR reviewed and edited the manuscript. All authors meet the ICMJE authorship criteria. All authors commented on the manuscript and approved the final version.

## Conflict of interest

The authors declare that the research was conducted in the absence of any commercial or financial relationships that could be construed as a potential conflict of interest.

## Publisher's note

All claims expressed in this article are solely those of the authors and do not necessarily represent those of their affiliated organizations, or those of the publisher, the editors and the reviewers. Any product that may be evaluated in this article, or claim that may be made by its manufacturer, is not guaranteed or endorsed by the publisher.
